# Python data odyssey: Mining user feedback from google play store

**DOI:** 10.1016/j.dib.2024.110499

**Published:** 2024-05-04

**Authors:** Affan Yasin, Rubia Fatima, Ahmad Nauman Ghazi, Ziqi Wei

**Affiliations:** a School of Software, Northwestern Polytechnical University, Xian, 710072, Shaanxi, China; b Department of Computer Science, Emerson University, Multan, Pakistan; c Department of Software Engineering, Blekinge Institute of Technology, SE-37179 Karlskrona, Sweden; d Institute of Automation, Chinese Academy of Sciences, Beijing, China

**Keywords:** Data mining, App reviews, User reviews, Crowd-source data, NLP

## Abstract

**Context:**

The Google Play Store is widely recognized as one of the largest platforms for downloading applications, both free and paid^1^. On a daily basis, millions of users avail themselves of this marketplace, sharing their thoughts through various means such as star ratings, user comments, suggestions, and feedback. These insights, in the form of comments and feedback, constitute a valuable resource for organizations, competitors, and emerging companies seeking to expand their market presence. These comments provide insights into app deficiencies, suggestions for new features, identified issues, and potential enhancements. Unlocking the potential of this repository of suggestions holds significant value.

**Objective:**

This study sought to gather and analyze user reviews from the Google Play store for leading game apps. The primary aim was to construct a dataset for subsequent analysis utilizing requirements engineering, machine learning, and competitive assessment.

**Methodology:**

The authors employed a Python-based web scraping method to extract a comprehensive set of over 429,000+ reviews from the Google Play pages of selected apps. The scraped data encompassed reviewer names (removed due to privacy), ratings, and the textual content of the reviews.

**Results:**

The outcome was a dataset comprising the extracted user reviews, ratings, and associated metadata. A total of 429,000+ reviews were acquired through the scraping process for popular apps like Subway Surfers, Candy Crush Saga, PUBG Mobile, among others. This dataset not only serves as a valuable educational resource for instructors, aiding in the training of students in data analysis, but also offers practitioners the opportunity for in-depth examination and insights (in the past data of top apps).

Specification Table

For enhanced clarity, the data specifications are presented in Table 1.

**Table 1:** Specification Table

Sr.No	Specification	Explanation
1.	*Subject*	Software Engineering, Computer Science Human-Computer Interaction
2.	*Specific Subject* *Area*	Requirements Engineering User Experience Research Marketing Analytics
3.	*Data Format*	XLSX / CSV file
4.	*Type of Data*	Table
5.	*Data Collection* *How data was acquired*	Data were extracted from Google Play store application page.
6.	*Data Source Collection*	The data was collected from two geographical locations e.g, Pakistan and China.
7.	*Code file*	python file (.py) for replication and extension.
8.	Dataset Accessibility (Game Apps)	1. Repository name: Zenodo 2. Link: https://zenodo.org/records/10029966
9.	*Dataset Accessibility* (Facebook App)	1. Repository name: Zenodo 2. Link: https://zenodo.org/records/10121935
10.	*Google Play store used for retrieval*	https://play.google.com/store/games*Detail links of games are given in the paper (See “Data Description” section).*

## 1 Value of the Data

• The following research areas demonstrate the potential applications of the extracted data:
  ­ *Requirements Engineering (RE):* Analyzing user reviews offers an empirical method for identifying customer preferences, complaints, and areas in need of improvement [1,2]. Through the consolidation of feedback from the user community, developers can methodically pinpoint features, modifications, and error corrections that cater to the most critical requirements of their intended user base. The utilization of user reviews from the Google Play Store is similarly observed in the field of Requirements Engineering (RE) as evidenced by the study [3].
  ­ *Machine Learning:* Utilizing sentiment analysis and natural language processing allows for the automated classification of review sentiment, the categorization of reviews based on topics, and the identification of frequently recurring themes [4]. These methods systematically unveil the distribution of customer sentiment, prevalent concerns, and requirements extracted directly from user feedback text, eliminating the need for manual inspection. The study [5] demonstrates a comparable application of user reviews from the Google Play store in the context of machine learning.
  ­ *Competitor Analysis:* Conducting competitive analysis by scrutinizing user feedback concerning competing applications can provide insights into market gaps and opportunities for differentiation that can be strategically pursued [6]. The study [7] showcases the use of user reviews for competitor analysis.
• A total of more than 429,000+ user reviews were extracted from the Google Play Store for various mobile applications. This extensive dataset serves as a valuable resource especially for the Requirements Engineering (RE) community, enabling the extraction of new requirements and the enhancement of applications based on user feedback, employing machine learning techniques for analysis and improvement.

## 2 Data Description

One of the reasons for selecting games under different categories is that they were among the top applications in their respective categories at the time of downloading. Furthermore, each CSV file is named after the corresponding application name. *The data collection took place in 2022.*

• We have extracted reviews for “Subway Surfers” mobile application developed by SYBO Games, which falls under the Free App category on the Google Play Store. To access the reviews please visit the provided link^2^: Subway Surfers on Google Play. • We have collected reviews for “Candy Crush Saga” mobile application developed by King, which is categorized as a Free App on the Google Play Store. To access the reviews please visit the provided link^3^: Candy Crush Saga on Google Play. • We have gathered reviews for “Rovio Classics: AB” mobile application developed by Rovio Entertainment Corporation, which is categorized as a Paid App on the Google Play Store. To access the reviews please visit the provided link^4^: Rovio Classics: AB on Google Play.
• We have obtained reviews for “Grand Theft Auto: San Andreas” mobile application developed by Rockstar Games, which falls under the Paid App category on the Google Play Store. To access the reviews please visit the provided link^5^: Grand Theft Auto: San Andreas on Google Play.
• We have collected reviews for “PUBG MOBILE” mobile application developed by Level Infinite, which is categorized under the Top Grossing section on the Google Play Store. To access the reviews please visit the provided link^6^: PUBG MOBILE on Google Play.
• We have compiled reviews for “Rise of Kingdoms: Lost Crusade” mobile application developed by LilithGames, which is listed under the Top Grossing category on the Google Play Store. To access the reviews please visit the provided link^7^: Rise of Kingdoms: Lost Crusade on Google Play.
• We have extracted and compiled more than 80,000 user reviews for the Facebook application from the Google Play Store. To access the reviews for Facebook, please visit the provided link^8^.
• Moreover, we have provided the Python script utilized for data extraction to benefit the research community. The script is accessible via the link provided in the specification table.
• The data template is displayed in Table 2. Furthermore, a detailed description of each attribute found in these files is presented below to enhance the reader's understanding.
  ­ *Rating:* It displays the user's rating, which can range from 0 to 5 stars.
  ­ *Reviews:* The following is a comprehensive textual review provided by a user.

## 3 Experimental Design, Materials and Methods

### 3.1 Data Extraction Process - Algorithm

• **Step1:** Initially, it is necessary to incorporate our URL into the Selenium web drivers.

Additionally, the essential libraries must be imported into the environment.

• **Step2:** In the second step, after accessing our website via the provided URL, we proceed to locate and interact with elements using Selenium. To identify these elements, various methods such as Xpath, CSS Selector, By ID, and By Name can be employed. Subsequently, each identified element is stored in separate variables. It's imperative to highlight the significance of implementing looping and page scrolling, which have been incorporated into the code.
• **Step3:** Once the elements are stored in variables, the subsequent step involves saving these variables to a CSV file.

**Table 2:** Dataset Attributes

Rating	Reviews	
4 stars	Great remake! However, I've noticed two glaring issues with it that should be fixed and may turn veterans away from the game. First, the physics don't quite feel like the original (e.g. Hal feels a little heavier and Red feels a little too light and isn't as bouncy). Second, many episodes that were present in the original game (e.g. Red's Mighty's Feathers, Bird Island, Short Fuse) are missing and I would like to see them added to the game. UPDATE: I'm glad that the sound is fixed!	

5 stars	Honestly a great remake, sure it doesn't have all of the episodes but they will probably add them in soon, aside from that it doesn't feel any different from the one I grew up playing, and as an extra plus it practically has no ads except for the ones that appear in the pause screen. I hope that the other games like space and seasons can get the same remake treatment	

4 stars	My kids and I have played this game for quite a few years and have really enjoyed it. Couldn't get it to work one day and that's when I realized it wasn't free any more if we wanted to conntinue to play so I thought yes definitely all our progress and it's fun.. so bought it and all the progress was wiped, gone!! Was not thrilled about that at all.. I would have just tried a different one if I knew our progress was going to be gone.	
.....		.....
.....		.....

**Fig. 1:**
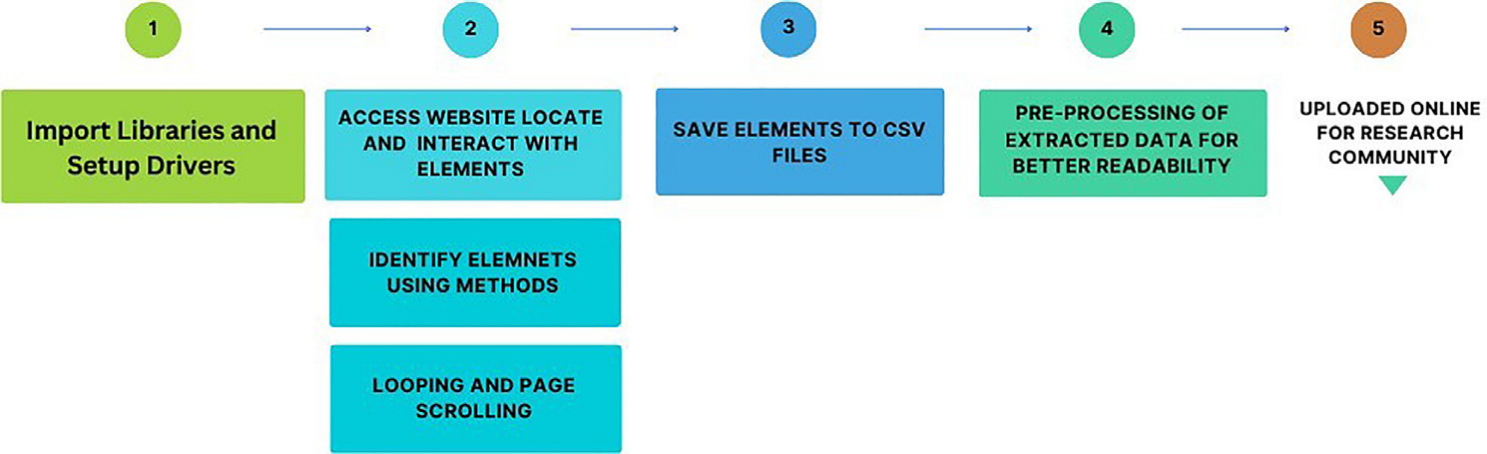
Visual Representation

The algorithmic steps are visually depicted in Fig. 1 to improve the reader's comprehension.

**Data Extraction Process - Detail Procedure:**

1. Setup Environment
  • Setup Environment (Python environment with Selenium installed (pip install selenium).
  • Initialize WebDriver
2. Navigate to App's Reviews Page:
  • Open the Google Play Store page for the target app.
  • Find and click the ”See all reviews” link.
3. Retrieve Reviews:
  • Wait for the reviews container to load.
  • Get the reviews container.
  • Iterate through the reviews and extract information.
4. Close the Browser:
  • Add a pause before closing the browser.

**Code Snippet:** The following code snippet is utilized to extract data from the Google PlayStoreforthechosenmobileapplications.

 

## Limitation

One limitation observed during the extraction process is that many users have employed various emojis in their reviews. In some instances, these emojis were not successfully captured, and instead, certain characters were displayed in the data-set.

## Ethics Statement

This study confirms that the current work does not involve human subjects, animal experiments, or any data collected from social media platforms.

• **Copyright:** Our project is committed to respecting copyright and intellectual property rights. We adhere to the legal and ethical standards for using content from the Google Play Store or applications. We only collect publicly available data and do not engage in activities that infringe on copyright laws. Any data we collect is used for research and analysis purposes only.
• **Privacy:** We prioritize user privacy and adhere to all relevant privacy laws and guidelines. When collecting data from the Google Play Store or applications, we ensure that no personally identifiable information is gathered. We focus solely on aggregating publicly available information while respecting user privacy and consent. Our data collection methods are in compliance with the applicable privacy policies and regulations.
• **Web Scraping Policies:** We are aware of the importance of respecting web scraping policies. For platforms like Google Play Store, we follow any specific scraping policies and guidelines they have in place. We use standard web scraping techniques and ratelimit our requests to minimize any impact on the platform. Our goal is to collect data responsibly, ensuring the integrity of the Google Play Store and applications.
• **Terms of Service:** We are committed to adhering to the Terms of Service (ToS) of the websites and platforms we interact with. This includes the Google Play Store and any applications we may access. We review and monitor the ToS regularly to ensure that our data collection practices remain compliant. If there are any specific scraping policies outlined in the ToS, we follow them closely.

## CRediT authorship contribution statement

**Affan Yasin:** Conceptualization, Methodology, Writing – original draft, Writing – review & editing. **Rubia Fatima:** Conceptualization, Methodology, Writing – original draft. **Ahmad Nauman Ghazi:** Software, Data curation, Visualization, Investigation, Writing – original draft, Writing – review & editing, Funding acquisition. **Ziqi Wei:** Software, Visualization, Validation, Formal analysis, Writing – review & editing.

## Data Availability

User Reviews - Mobile Game Apps (Original data) (Zenodo).

## Footnotes

2 https://play.google.com/store/apps/details?id=com.kiloo.subwaysurf.

3 https://play.google.com/store/apps/details?id=com.king.candycrushsaga.

4 https://play.google.com/store/apps/details?id=com.rovio.abclassic22.

5 https://play.google.com/store/apps/details?id=com.rockstargames.gtasa.

6 https://play.google.com/store/apps/details?id=com.tencent.ig.

7 https://play.google.com/store/apps/details?id=com.lilithgame.roc.gp.

8 https://play.google.com/store/apps/details?id=com.facebook.katana.
